# The death of the Empress Elisabeth of Austria and Queen of Hungary—retold and reassessed with reconstruction of her autopsy

**DOI:** 10.1007/s10354-024-01042-y

**Published:** 2024-05-17

**Authors:** Roland Sedivy

**Affiliations:** 1 Chair of Clinical Pathology und Molecular Pathology, Sigmund Freud University Vienna, Vienna, Austria; 2 Institute of Pathology, Cytology und Microbiology Dr. Kosak GmbH, Mariannengasse 14, 1090 Vienna, Austria

**Keywords:** Empress Elisabeth of Austria, Sisi, History of medicine, Autopsy, Assassination, Celebrity, Kaiserin Elisabeth von Österreich, Sisi, Geschichte der Medizin, Autopsie, Ermordung, Prominente

## Abstract

In this publication 125 years after the violent death of the famous Empress Elisabeth (1837–1898) of Austria, known as Sisi, a modern evaluation as well as valuation will be presented. The day after her assassination by the young anarchist Luigi Lucheni using a file, a partial autopsy was performed to find out the exact cause of death—a pericardial tamponade. The complete translation of the autopsy report is part of this article, the intention being to avoid unclear wording and translation errors, which have often caused some confusion in the past. A pictorial illustration of the puncture canal will provide clarity through medical facts as to how Empress Elisabeth’s death came about by correct pathological and anatomical description and, thus, counteract former interpretations.

## Abstract (de)

Vor 125 Jahren wurde die berühmte Kaiserin Elisabeth (Sisi, 1837–1898) in Genf durch den Anarchisten Luigi Lucheni mit einer Feile ermordet. Am nächsten Tag wurde Sisi obduziert und eine Herzbeuteltamponade als Folge der Herzverletzung festgestellt. In dieser Publikation wird der gesamte Autopsiebericht abgedruckt und einer modernen Interpretation und Bewertung aus heutiger Sicht unterzogen. Die Absicht dessen ist, unklare Formulierungen und Übersetzungsfehler zu vermeiden, die in der Vergangenheit oft zur Verwirrung geführt haben. Zudem wurde der Stichkanal bei einer nachgestellten Obduktion rekonstruiert, um auch frühere widersprüchliche Interpretationen zu klären.

## Introduction

**Fig. 1:**
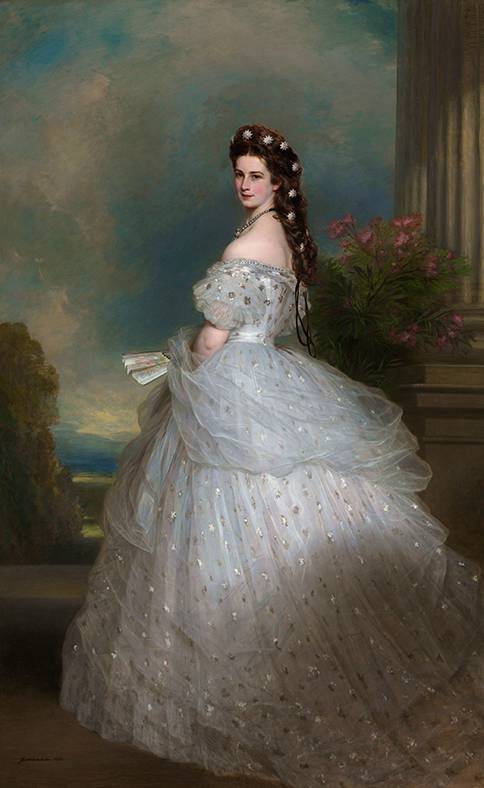
Empress Elisabeth in ballgown with diamond stars in her hair. (Painting by Franz Xaver Winterhalter, 1865, oil on canvas KHM Vienna [Wikipedia; CC0 1.0 DEED])

Elisabeth (1837–1898), Empress of Austria and Queen of Hungary (Fig. 1), was a tragic figure in the late history of the Austrian Empire. She was born on 4 December 1837 in Munich, as Princess of Wittelsbach, Duchess in Bavaria, the fourth of ten children of Duke Maximilian in Bavaria (1808–1888) and his wife Maria Ludovika (1808–1892), who happened to be the sister of Emperor Francis Joseph’s I mother, Sophie. These children were raised in relative freedom, far away from any strict court ceremonial. Firstly, her sister Helene, called Néné (1834–1890), was intended to marry Francis Joseph of Habsburg-Lothringen. However, as it happens in life, he fell in love with Elisabeth when she accompanied her sister to an arranged meeting in Bad Ischl. Francis Joseph decided to marry the not even 16 years old Elisabeth on 24 April 1854 in Vienna. The strict conventions of the Viennese court and her extremely conservative mother-in-law and aunt came as a big shock to the shy and immature young Elisabeth. Although the two young people seemed to be in love, her main duty and role was to produce predominantly a male heir. More and more she suffered under the rules that did not allow any privacy. The couple had four children, Sophie (1855), Gisela (1856), Rudolf (1858), and, the most loved one, Marie Valerie (1868)—so Elisabeth went through three pregnancies before her 21st birthday, lastly 10 years later, she gave birth to Valerie. Because she was said to be unfit to raise her first three children, her mother-in-law and governesses took over. Unfortunately, when she insisted on taking her two little daughters on a journey to Hungary in 1857, the 2‑year-old daughter Sophie died, presumably of typhoid fever.

Thus, Elisabeth, who had been pressed into the role of an empress, remained alien all her life. Mostly surrounded by people who bullied her, she became increasingly exhausted and depressed. She tried to avoid the Viennese court by travelling most of the time, spending her time away in Korfu, Madeira, and Greece. In all its tragedy, in 1889, her son Rudolf committed suicide at Mayerling, shooting first his love affair Mary Vetsera and then himself.

**Fig. 2:**
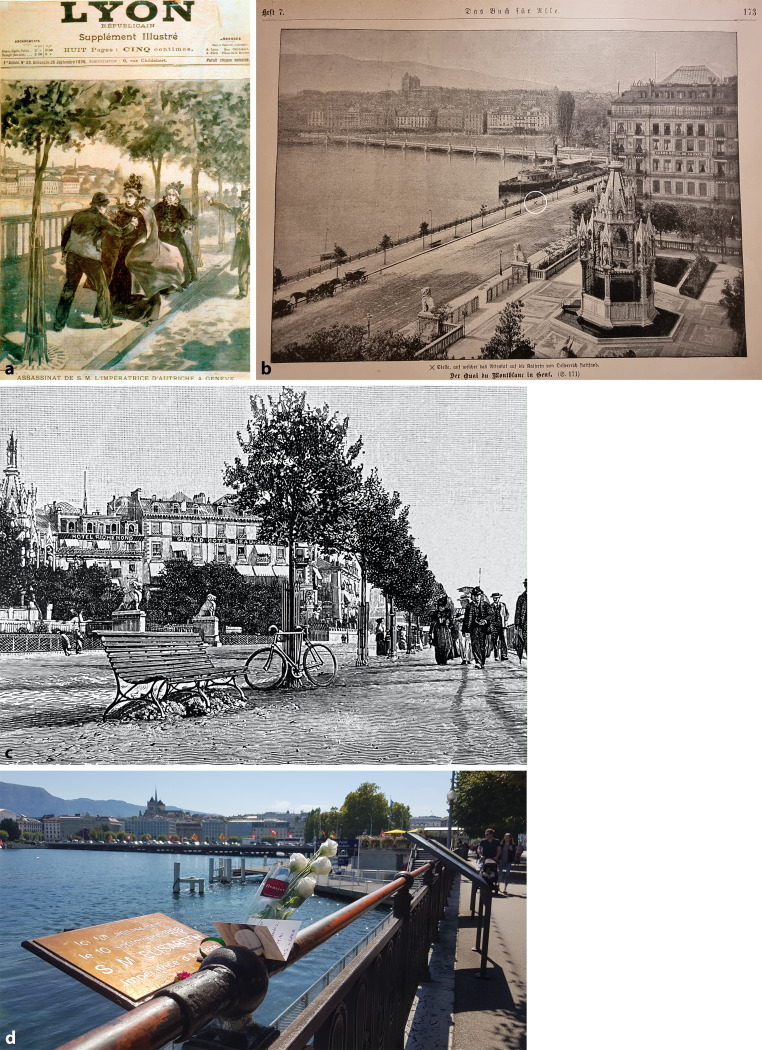
Crime scene Quai du Mont-Blanc. **a** Newspaper illustration, artistic frontpage of the *Lyon republicain*, *Supplement illustre*, 25. Sept. 1898; 18980925_PD0005—akg-images/picturedesk.com, bird’s eye view—*circle* indicates location of the attack; **b** reprint of photography, collection Sedivy; **c** crime scene up close, clu, iStock by Getty Images illustration-ID:1296163148; **d** nowadays (2018), photography by R Sedivy

**Fig. 3:**
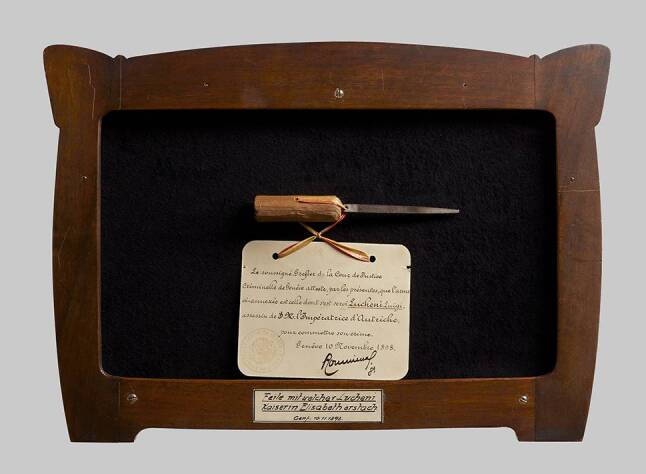
Murder weapon (the sharpened file is kept today at the Josephinum in Vienna). (Photo: Bene Croy. Source: Josephinum, Ethics, Collections and History of Medicine, MedUni Vienna)

Elisabeth traveled a lot, probably to escape predominantly the strict court in Vienna and to overcome their strokes of fate [1–4]. Her tragic end came on one of her journeys on 10 September 1898, when the Empress was visiting Geneva, Switzerland. Although she mostly travelled under the false name “Countess of Hohenembs,” the tabloids reported her presence and fate took its course. The 25-year-old Luigi Lucheni, an Italian anarchist, decided to go down in history by killing the Empress. Primarily, he wanted to kill Prince Henri of Orléans, but the noble left Geneva earlier than expected. In Lausanne, Lucheni read in a newspaper that Empress Elisabeth had arrived surprisingly in Geneva. For Lucheni it was much more significant to assassinate the Empress of Austria. Because he did not have enough money for the intended revolver or dagger, he bought a triangular, sharpened file. His buddy Gabriel Martinelli mounted a wooden handle on it and the weapon was thus ready. When Elisabeth, together with her lady-in-waiting, Countess Irma Sztáray, walked along the Quai du Mont-Blanc to board an excursion boat, Lucheni attacked her with that sharpened triangular file ([5]; Figs. 2 and 3).

## The assassination

Lucheni said at the police interview, “As the two of them stepped out onto the street, I was leaning on the railing of the lakeshore. I ran towards her and blocked her way. I bent down and looked under the umbrella. I didn’t want to get the wrong one. They both wore black. She wasn’t particularly pretty. Quite old already.”

“When I gave her the push, I knew she was going to die. I pushed with all my strength and felt the weapon penetrate deep into her chest. She also fell over as if struck by lightning” [6].

**Fig. 4:**
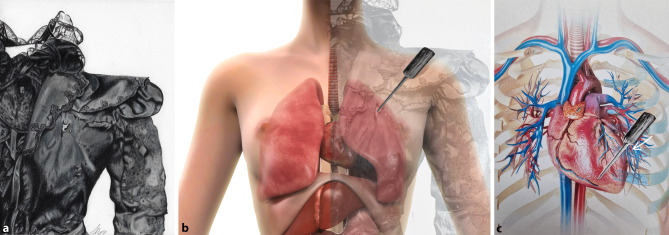
Graphical reconstruction. Blouse with puncture (**a**), drawing according to photography by Mrs. S. Rupp vulgo Madam Nadel. **b** Overlay of the death blouse with anatomical torso, torso basic image by leonello, iStock by Getty Images, stock-ID:1140422390, postprocessing with overlays by R. Sedivy; **c** superimposition of the file with the thoracic organs and ribs, whereby the *marked point* shows the impingement of the shaft on the 4th rib, which was fractured by it, torso basic image by leonello, iStock by Getty Images, stock-ID:1140421171, post-processing with overlays and drawing by R. Sedivy

Lucheni thrust his right arm at a steep angle from top right to bottom left, and pierced the skin 11 cm beside the sternum and 11 cm below the clavicle (Fig. 4). It happened so quickly that the Empress was not even aware of what had happened. After the blow to her chest, Sisi stood up, thinking she had only been punched by the stranger. No one saw any weapon, and it was thought that Lucheni might have intended to steal something. Elisabeth asked, “What do you think the man wanted from me? Maybe my watch?” [7].

Still on the Quai du Mont-Blanc, the bell of the steamship Genève rang to warn of its imminent departure. The Empress and her lady-in-waiting were only a few yards away and rushed quickly on board. At the jetty Elisabeth felt dizzy and held on to her lady-in-waiting. Few minutes later, on the deck of the ship, the Empress turned very pale and lost consciousness. A small reddish-brown stain the size of a coin was found on her undershirt after the bodice was opened. The captain was informed of the illness and true identity of his passenger. Thus, the steamboat immediately turned back to port. She then briefly regained consciousness and said her famous last words: “What actually did happen to me?” Again unconscious, the Empress Elisabeth was carried back to the Hôtel Beau-Rivage by six sailors on an improvised stretcher, where she arrived at 2:15 pm. An immediately called practitioner, Dr. Etiènne Golay, and shortly after him Dr. Mayor followed by a priest, arrived at the hotel room number 34 in the first floor. Fanny Mayer, the wife of the hotel’s director, a visiting nurse, and the lady-in-waiting Countess Sztáray undressed the Empress and removed her shoes. When they then removed her from the stretcher to the bed, Mrs. Mayer believed to hear two audible breaths—maybe the last ones [8]. Dr. Mayor incised the artery of her left arm but there did not follow any remaining blood flow. Allegedly, to ascertain death, Dr. Mayor tapped with a reflex hammer around and on the nipple—this surprising approach was mentioned in an informal talk between prosecutor and both doctors in the police report.

Empress Elisabeth of Austria and Queen of Hungary was pronounced dead at 2:10 p. m. A partial autopsy was performed at 2 o’clock next day and after 50 min a pericardial tamponade was found as cause the of death due to the file wounds.

## Official documents

**Fig. 5:**
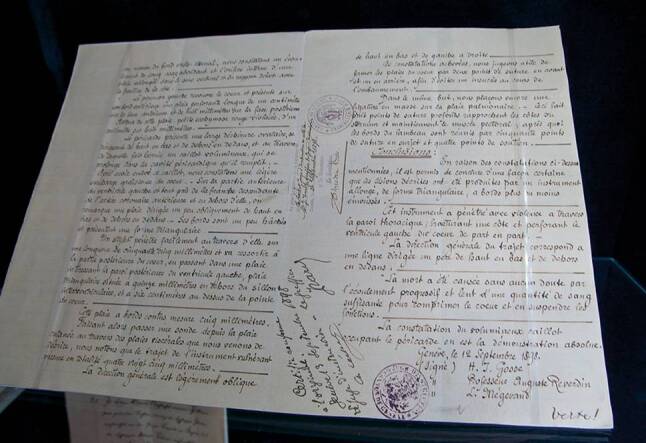
Two pages of the autopsy report. (Wikipedia; CC0 1.0 DEED)

There are two reports stored at the Austrian State Archives comprising the order of the postmortem examination by the examining judge, the compilation of the medical commission as well as the external inspection and description of the body, carried out on the afternoon of 10 September 1898 (Fig. 5).

### First document—external description

“I, the last person to sign, Louis J. A. Mégevand, Doctor of Medicine, Associate Professor of Forensic Medicine at the Medical Faculty of Geneva, Assistant to the Laboratory of Forensic Medicine of the said Faculty, was commissioned by the Honorable Examining Judge of the Republic of the Canton of Geneva to carry out an examination and this order was received by four o’clock in the afternoon of September 10th, 1898:

The examining judge of the Republic of the Canton of Geneva commissions Dr. L. Mégevand, to create a medical report on the passing of Her Majesty the Empress of Austria; the cause of death and, if necessary, to perform an autopsy in agreement with the Empress’s heir or heirs.”

[Note from author: Francis Joseph I did not want an autopsy, but he replied that one should proceed according to the laws of the country.]

“Dr. Mégevand brings in one or two colleagues, professors from the faculty, to assist.”

Previous swearing

“I declare that on September 10, 1898, at 5 o’clock in the afternoon, I went to the Hotel Beau-Rivage on the Léman quay for the purpose of examining Her Majesty’s body. I underwent this examination in the presence of Dr. Auguste Reverdin, professor at the Medical Faculty in Geneva, who was also appointed by the public prosecutor’s office. Professor Gosse, who was absent from Geneva, was unable to take part in this first part of the investigation. Dr. Etiènne Golay, who has rendered services to Her Majesty, was authorized by the examining magistrate to be present at this examination.

On September 10, 1898 at 5 p. m., when we arrived at the Hotel Beau-Rivage, we entered room number 34, which is on the first floor and faces the Léman quay. In this room lay stretched out on a bed the body of a woman, which Countess von Sztáray described to us as that of Her Majesty Elisabeth-Amalia-Eugenie, Empress of Austria, Queen of Hungary. The body was completely naked and covered with a bed sheet. We are now moving on to examining the body. This is that of a woman of about 60 years, with the following description:

The external impression is calm, apparently without muscle spasms. The skin is loose and there is no rigor mortis. The skin color is yellowish-pale. The hair is dark brown. The eyes are grey-blue. Good teeth.

The subcutaneous tissue is poorly developed. The height is 1 meter 72. On the abdomen pearly, old welts.

There are already some signs of dead spots on the dependent parts. We don’t find any leakage from the nose or mouth.

We note the following injuries on the left front and side wall of the thorax:

Eleven cm below the left clavicle and eleven cm outside the median line passing through the sternum, there is a wound in the shape of a ‘V’ on the left side and four cm above the nipple, with the opening facing upwards and outwards. The upper branch, which extends from top to bottom and slightly from left to right, measures 1 centimeter in width. Another branch, 1.2 cm long, arises from its lower part in an almost horizontal direction. The edges of the vertical branch show a gap of 2 mm, [note from author: the measurements have been adapted to modern notation.] the horizontal piece has a gap of 4 mm. The wound itself has a dried and brownish appearance; their edges are surrounded by a small, bloodshot, slightly bluish zone a few millimeters wide.

There were some injuries around the nipple and in its vicinity, which were reported to us by Doctors Golay and Mayor as a result of the use of the reflex hammer were declared by Mayor. The very superficial injuries seem to us to agree with our colleagues explanation.

The right wrist is wrapped with a strip of hydrophilic gauze stained with bloody fluid. After this strip has been removed, we notice a small wound on the lower part of the radial region, the edges of which are clearly demarcated in a straight line and which runs parallel to the axis of the limb and corresponding to the course of the radial artery. This wound measures about 2 cm in length and was made post-mortem, according to our colleagues, for the purpose of determining death. Their edges were united by two button seams.

In witness whereof we have signed this report.

Geneva September 12, 1898

(signed) L. Mégevand.

(signed) Professor Auguste Reverdin.”

This handwritten text was then certified as true to the original by the investigating judge and the clerk who appended their signatures.

### Second document—autopsy report

“We, the undersigned Hippolyte Jean Gosse, Doctor and Professor of Legal Medicine at the University of Geneva, Auguste Reverdin, Doctor and Professor at the Faculty of Medicine of Geneva, and Louis Mégevand, Doctor and Privat Docent at the Faculty of Medicine of Geneva, were requested on September 11, 1898 by Mr. Lèchet, investigating Judge of the Republic and Canton of Geneva, to carry out the partial autopsy of HM Empress Elisabeth of Austria in order to determine with certainty the causes of her death.

We carried out this investigation on September 11, 1898 at half past two in the afternoon, in the presence of Madame Countess Sztáray, Lady of Honor to Her Majesty, the Count of Kufstein, consul of Austria in Berne, General Berzeviczy, delegate of His Majesty, Mr Navazza, Prosecutor General of the Republic and Canton of Geneva, Mr Dr Mayor, professor at the Faculty of Medicine of Geneva and Mr Dr Etienne Golay; the latter two having given care to Her Majesty.

An incision was made from the outer edge of the sternal region, extending 43 cm outward in a concave line. This line delineates a window-like flap that affects all of the soft tissues covering the left-sided thorax.

This flap remains attached to its external part and allows the ribs and the intercostal muscles to be viewed and a penetrating wound to be discovered which is in direct connection with the soft tissue wound already described in the previous external description.

This triangular wound has a side length of eight millimeters. It lies eleven cm from the median line and passes through the third intercostal space to the fourth rib, which splits it across its entire thickness.

One notices an all-encompassing bloodshot around this wound. If we lift up the thoracic wall, which we have divided in the region of the costal sternal border, we find a fairly copious discharge of blood and the internal opening of a wound extending vertically and in direct relation to the rib fracture.

The left lung covers the heart and shows a perforating wound on its front edge, one centimeter long on the front of the lung and eight millimeters long on the back of the lung. Around this wound there is a small bloodshot of red-purple color, measuring one centimeter by eight millimeters.

The pericardium shows a wide oval tear that extends from top to bottom and outside to inside. Across it is a herniated, voluminous blood clot, which extends into the pericardial space and fills it. After this blood clot has been removed, we notice a small amount of fatty overgrowth in the heart. Above the anterior portion of the left ventricle, and very close to and outside the descending branch of the anterior coronary artery, one notices a wound running somewhat obliquely from top to bottom and from outside to inside. Their edges are a little chopped and offer a triangular shape.

A fine probe (‘un stylet’) easily penetrates this wound to a length of 55 millimeters and emerges from the back of the heart. It passes through a three-angled wound that affects the posterior wall of the left ventricle and is 15 millimeters outside the interventricular groove and 6 cm above the apex of the heart.

This wound has bruised edges and measures five millimeters.

After passing a probe through the skin wounds and the internal wounds which we have just described, we note that the distance of the wounding instrument in its entirety measures eighty-five millimeters.

The general direction [note from the author: of the wound channel] is oblique, from top to bottom and from left to right.

Having completed these findings, we find it useful to close the heart wounds front and back by two button sutures, in order to avoid failure in the course of embalming.

For the same purpose we also place a ligature in the area of the lung wound. After this has been done, we join the sternal ribs together with eight deep button sutures and unite the pectoral muscles; afterwards the edges of the flap are united by fifty continuous overcast sutures and four fixation sutures.

Conclusions:

Taking into account the above findings, it is permissible to conclude with certainty that the injuries described were caused by a long instrument of triangular shape with more or less blunt edges. This instrument penetrated forcefully through the chest wall; it fractured a rib and perforated the left ventricle through and through. The general direction of the wound channel corresponds to line that runs slightly from top to bottom and from outside to inside.

Death was undoubtedly caused by the progressive and slow outflow of such a quantity of blood that compressed the heart and suspended its functions.

The finding of an extensive blood clot filling the pericardium is the absolute proof of it.

Geneva, September 12, 1898.

(Signed) H.J. Gosse

–^II^– Professor Auguste Reverdin

–^II^– L. Mégevand*”*

## Pathoanatomical reconstruction

An attempt was made to reconstruct the course of the assassination based on the autopsy report. The French doctors found a stab wound in the anterior chest wall of 8.5 cm in depth, the file measured 10 cm. During the police interview, Lucheni stated that he hit with full force and noticed that his tool penetrated deeply into the Empress. The file had penetrated the third intercostal space, pierced the anterior part of the lung and the heart on the left anterior and posterior wall. With the 2 cm wide shaft of the file, he hit the fourth rib, which was broken by such a forceful impact.

**Fig. 6:**
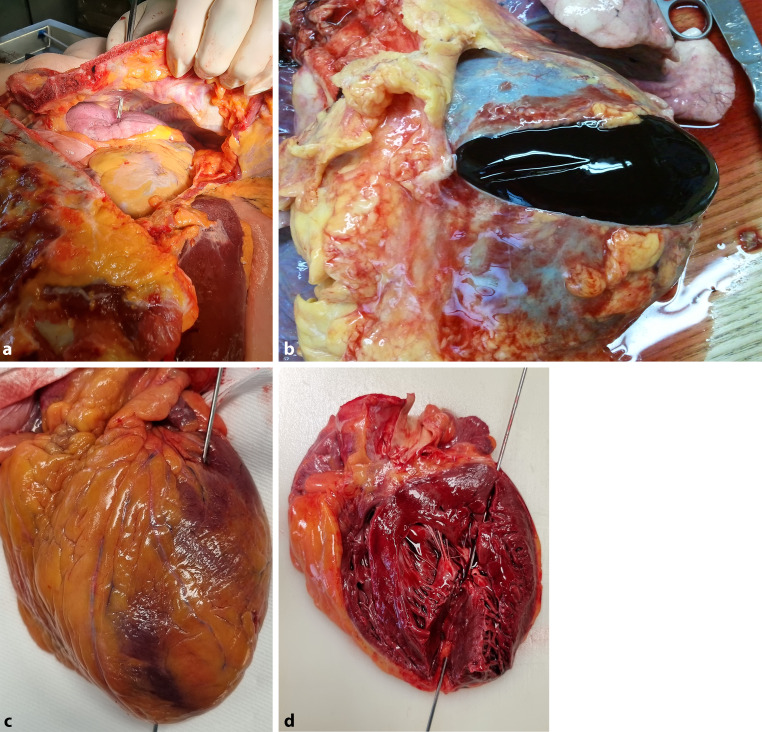
Autopsy reconstruction. **a** Puncture channel through the chest with the lungs, **b** the pericardial tamponade, **c**, **d** puncture channel through the heart. (All images a–d by R. Sedivy)

To illustrate the tracks of the stab wounds, superimposed images of the body, the excised organ, and a knife model were constructed to obtain a 2D model. This allowed clear and concise visualization of the complex relationship of the knife to the heart incision and the stab wound on the chest surface (Fig. 6).

## New evaluation and valuation of her death

Empress Elisabeth died due to pericardial tamponade caused by stab wound using a file. Such instruments are rarely used. In the literature, a rare example was published where a screwdriver was used [9]. Nevertheless, although the weapon is very unusual, such myocardial wounds are well known. Cardiac wounds have been described for centuries and still remain fatal. In addition, for a long period of time, the suturing of a myocardial laceration was thought to be very difficult and, even nowadays, two cardiac stab wounds need emergency surgery [10]. Meyer et al. argued that the life of Empress Elisabeth could have been saved because “the physiology and clinical signs of cardiac tamponade were already well known. Pericardiocentesis, the emergency treatment, had been performed for more than half a century, and the first myocardial suture had just been successfully attempted 2 years earlier” [11]. I doubt this because the rescue chain at that time was not comparable to that of today. And even today, cardiac tamponade is only survived in exceptional cases. In general, if the effusion forms quickly, the critical amount of exudate is approximately 300–400 ml. An acute iatrogenic hemorrhage of just 100 ml can lead to life-threatening pericardial tamponade. If pericardial tamponade develops slowly, the amount of fluid may increase to more than 2000 ml. For instance, an aortic dissection or a rupture in myocardial infarction as a cause of pericardial tamponade ends regularly quickly fatally [12–14].

It is claimed that Lucheni skillfully—and with very good knowledge of anatomy—stabbed the empress specifically in the thorax [1]. I deny this, because he quickly walked towards the Empress, looked shortly under her umbrella to see her face, and smashed his fist from top to bottom onto her left chest. The Empress had a high-necked black blouse, thus precise positioning was not possible in that hurry. A day before he observed both ladies during a short walk out of the hotel to be sure who the Empress was. In the interrogation protocol, he said that Elisabeth looked rather old and, thus, he just had a short look under her umbrella to hit the right person, because both ladies were dressed black.

In addition, there is no indication in his biography nor in the interrogation protocols that he might have acquired anatomical knowledge.

Another issue of discussion was the delay in the onset of the first symptoms. Because of the sharpness and thinness of the file, the wounds were very narrow. We can therefore assume that the sharp-edged wound in the myocardium pumped only a few spurts of blood into the pericardium with each systole. Unlike irregular, frayed heart wall ruptures, e.g., caused by a heart attack, the slit-shaped wounds of the pointed file only allowed a small volume of blood to pass through. Nevertheless, the progressive accumulation of blood leaking from the double perforation of the left ventricle into the pericardial space led at its end to a high mechanical pressure, thus resulting in an important decrease in stroke volume and leading to severe arterial hypotension. This explains the dizziness on the jetty followed by the first syncope on board.

It is often discussed whether the tight lacing of her corsets and bodice slowed down the bleeding and whether opening it might have hastened her death. The answer is definitely no! Firstly, the skin wound was not the problem and, secondly, the thorax was not so compressed by the bodice that the bleeding from the heart into the pericardium could have been influenced by it! The bleeding into the pericardial sac out of the heart chamber was slowed to mere drops due to the sharp wound edges. Not until the sac filled was the beating of her heart really impeded, which is why the Empress was able to walk from the site of the assault up to the steamer’s ramp. If the weapon had not been removed, she would have lived a while longer, as it would have acted like a plug to stop the bleeding.

Were there other illnesses that contributed to the Empress’ violent death? Definitely not!

The “court recipe books” kept by the Austrian State Archives provide fairly precise information about the medicines that were mixed and brewed in the pharmacy at the Vienna Hofburg for the high-ranking ladies and gentlemen [15]. What conclusions could be drawn from this about health problems of Empress Elisabeth?

There were a wide range of ointments, tinctures, and drinks prepared, which might indicate a number of ailments. But they were of psychosomatic origin and do not support in any way the violent death of the Empress Elisabeth. Most noticeably, she cultivated a cult of beauty that cost her and her servants several hours a day. The Empress, who had given birth to four children, was able to maintain a waist measurement of 50 cm well into her old age, which was only possible by wearing a corset and sewing it into tight clothes. Fasting cures, combined with excessive exercise, should have led to health damage, such as hunger edema, which should be compensated with medication. In her writings and biographies there is mentioned a kind of rheumatism and sciatica. What is meant in this context is certainly not medical rheumatism, but rather the so-called soft tissue rheumatism. It is also said that she had a tendency to melancholy, sometimes even interpreted as depression. As a strong remedy to overcome that depression, cocaine had been administered intravenously to Elisabeth. This because the drug became a sought-after substance among the better circles, since Sigmund Freud recommended this drug in 1884 as a miracle cure for depression, impotence, and other ailments.

What is missing or unclear in the original report?

It is rather surprising that the external examination was that early, although rigor mortis had not yet occurred. At those times, the examination of the body for dead spots and rigor mortis were already common practice to certify death. However, the bloodletting was performed for certification of the occurred death because no blood flowed from the arm artery. There were also some injuries around the nipple and in its vicinity, which were reported to us by Doctors Golay and Mayor as a result of the use of the reflex hammer. In the last century, it was said that the French tested death by tapping the nipple with a reflex hammer [16]. In my opinion this action is rather strange and unusual. Was it an attempt to create brown dry spots? Even the adhesive pads from an EKG can cause reddening of the skin in the living and drying out in the dead. But this is not a reliable sign of death!

Eventually, there is no mention of a tattoo in the autopsy report, even though it is often claimed. However, we know this from the diary of her favorite daughter Marie Valerie, who mentions that she saw an anchor on her mother’s back [17].
