# Erratum zu: The death of the Empress Elisabeth of Austria and Queen of Hungary—retold and reassessed with reconstruction of her autopsy

**DOI:** 10.1007/s10354-024-01048-6

**Published:** 2024-07-09

**Authors:** Roland Sedivy

**Affiliations:** 1 Chair of Clinical Pathology und Molecular Pathology, Sigmund Freud University Vienna, Vienna, Austria; 2 Institute of Pathology, Cytology und Microbiology Dr. Kosak GmbH, Mariannengasse 14, 1090 Vienna, Austria

**Erratum zu:**

**Wien Med Wochenschr 2024**

10.1007/s10354-024-01042-y

Unfortunately, there are 2 numerical errors in the article:

Introduction 2nd line: Elisabeth is born on December 24th – and not on December 4th!

On page displaying Fig. 2: left column, paragraph 2. line 2: … was pronounced dead at 2:40 p.m. – not at 2:10 p.m.

